# Psychometric validation of the Functional Assessment of Cancer Therapy‐Endometrial among endometrial cancer patients

**DOI:** 10.1002/cam4.7096

**Published:** 2024-03-11

**Authors:** Sooyeon Kim, Joseph J. Noh, Youngha Kim, Juhee Cho, Danbee Kang, Yoo‐Young Lee

**Affiliations:** ^1^ Center for Clinical Epidemiology Samsung Medical Center Seoul Korea; ^2^ Department of Clinical Research Design and Evaluation, SAIHST Sungkyunkwan University Seoul Korea; ^3^ Department of Obstetrics and Gynecology, Samsung Medical Center Sungkyunkwan University School of Medicine Seoul Korea

**Keywords:** endometrial cancer, patient‐reported outcome, quality of life, symptom, validation

## Abstract

**Objective:**

To evaluate a psychometric validation of the endometrial cancer subscales (EnCS) in the Functional Assessment of Cancer Therapy‐Endometrial (FACT‐EN) among patients with endometrial cancer.

**Methods:**

This cross‐sectional study was conducted at a tertiary university‐based hospital in South Korea between April and October 2022. Participants completed a survey questionnaire that included the FACT‐EN. Exploratory and confirmatory factor analyses (EFA, CFA) and the reliability were measured using the intraclass correlation coefficient (ICC) under a two‐way mixed model. Pearson's correlations were used to evaluate the validity. We also tested known‐group validity.

**Results:**

In total, 240 patients with endometrial cancer participated in the survey. In EFA, we found EnCS included four domains. In CFA, four‐factor solution model was good: CFI = 0.659; SRMR = 0.066, and RMSEA = 0.073. The mean (SD) of total score of FACT‐EN was 122.84 (23.58). The floor and ceiling effects were 0.4% and 0.4%, respectively. Cronbach's *α* coefficients for the five scales of the EnCS ranged from 0.78 to 0.91. The ICC of EnCS was 0.76. The convergent and discriminant validity of EnCS was acceptable. In the group analysis, older age and lower ECOG performance scores were associated with higher EnCS scores. The stomach and vaginal domains in EnCS were higher in patients who had completed treatment for more than 1 year compared to those who were still undergoing treatment.

**Conclusions:**

FACT‐EN has demonstrated its validity as an assessment tool with significant implications for capturing various symptoms in patients with endometrial cancer.

## 1 BACKGROUND

Endometrial cancer (EC) is the most commonly diagnosed gynecological cancer among women in North America and Europe.^1^ Worldwide, endometrial cancer ranks as the sixth most common malignancy in women, and its incidence and mortality rates are increasing.^1^, ^2^ Comprehensive surgical staging plays a crucial role in guiding the decision for adjuvant therapy such as vaginal brachytherapy and external beam radiotherapy^3^; however, it is important to consider the potential adverse effects of these treatments, such as long‐term toxicities (e.g., sexual, digestive issues with radiation, hematological and neuropathy‐related complications with chemotherapy).^4^, ^5^, ^6^ Those negative effects from treatment might generate poor quality of life (QoL). Addressing QoL during cancer treatment is a critical component because it contributes to patients' outcome and overall well‐being.^7^ In addition, healthcare providers can use the QoL measure to gain insight into how the disease and its treatment affect patients' daily functioning, symptoms, and overall satisfaction with life. QoL measures also provide a comprehensive understanding of the patient's experiences beyond traditional clinical outcomes.

QoL among patients with endometrial cancer is often assessed using the European Organization for Research and Treatment of Cancer Quality of Life Questionnaire‐Core 30 (EORTC QLQ‐C30), Short Form‐36 (SF‐36), and the Functional Assessment of Cancer Therapy‐General (FACT‐G).^6^ While these QoL measures addressed important parameters that patients with endometrial cancer experience, they did not cover unique symptoms, such as urological and sexual symptoms.^6^ Thus, researchers have used additional symptom‐specific and endometrial cancer‐specific QoL measures, including the Female Sexual Function Index (FSFI), the European Organization for Research and Treatment of Cancer Quality of Life Questionnaire—Endometrial Cancer module (EORTC QLQ‐EN24), and the Functional Assessment of Cancer Therapy‐Endometrial (FACT‐EN).^6^, ^8^

Among these tools, the EORTC QLQ‐EN24 and FACT‐EN were the most commonly used questionnaires.^8^ Yet, EORTC QLQ EN24 did not include menopausal symptoms which is a frequent problem among endometrial cancer patients. In contrast, the FACT‐EN includes menopausal‐like symptoms such as hot flushes and vaginal discharge; thus, it is more widely used as a QoL measure among this patient population.^9^ In fact, the FACT‐EN included unique features, and the problem among patients with endometrial cancer,^10^ and it is necessary to have validated tool. However, there was lack of worldwide validation, specifically for EnCS (source: https://www.facit.org/measures/FACT‐En) of FACT‐En. In this study, we conducted a validation study of the EnCS within the FACT‐EN measure in patients with endometrial cancer.

## 2 METHOD

### 2.1 Study participants

Although a global validation study was not conducted, we performed a validation study in Korea. Korea has a stable healthcare system with widespread access to medical care. We expected that Korea validation study provides a diverse and representative sample of patients for studies, increasing the validity of the results. Thus, this cross‐sectional study was conducted at a tertiary university‐based hospital in South Korea between April and October 2022. We included women (1) aged above 18 years, (2) diagnosed with endometrial cancer, (3) who were planning to or receiving treatment, or who had completed treatment within 6 months, and (4) who were able to speak and read Korean. We excluded patients who (1) had secondary cancer, metastasis, or recurrence, or (2) had any physical or psychiatric conditions that would interfere with completing the questionnaire.

### 2.2 Ethical approval

This study was approved by the Institutional Review Board of the Samsung Medical Center, Seoul, Republic of Korea in the development set (IRB No. SMC‐2021‐08‐117). Informed consent was obtained from all study participants.

### 2.3 Measurement

The FACT‐EN was developed by the Functional Assessment of Chronic Illness Therapy multilingual translation (FACITtrans) to measure the function and symptoms of patients with endometrial cancer. The FACT‐EN consists of 43 items with five domains: physical well‐being (PWB), social well‐being (SWB), emotional well‐being (EWB), functional well‐being (FWB), and endometrial cancer subscale (EnCS). The PWB, SWB, EWB, and FWB were obtained from the FACT‐G and have already been validated in the Korean population.^11^ The EnCS consists of 16 items relevant to endometrial cancer treatment: gastrointestinal, vaginal, and urological symptoms; body image; fatigue; musculoskeletal pain; and taste changes. The patients were asked to rate how they felt during the past 7 days. All FACT scores used a 5 points Likert‐type response scale and were scored in accordance with the guidelines provided by factit.org, with high scores representing better QOL. The ranges of scores for these scales were 0–28, 0–28, 0–24, 0–28, and 0–64 for the five domains, respectively, and the range of the total score for the FACT‐EN was 0–172. A higher score indicates a higher quality of life. The Korean version of the FACT‐EN used in this study has been translated and linguistically validated by FACITtrans.^12^ However, the FACIT Group only translated FACT‐EN and did not perform psychometric validation. Therefore, we confirmed with the FACIT Group that a validation study using the Korean translation version among patients with endometrial cancer would be performed. Subsequently, we obtained copyright permission from FACITtrans.

Before conducting the cross‐sectional survey for psychometric validation, we conducted cognitive interviews to confirm the content validity of the scale using the standardized methodology developed by FACITtrans. The eligibility of the study participants for the qualitative interviews was the same as the criteria for the cross‐sectional survey. Participants were asked to complete a questionnaire and subsequently underwent a 30‐min cognitive debriefing to evaluate their comprehension, ease of response, and acceptability of the terminology, phrasing, and response options. The interviews were conducted by a trained behavioral scientist. We recruited study participants until saturation.

To evaluate convergent and discriminant validity of the EnCS, we used the Korean version of the EORTC QLQ‐EN24,^13^ the Menopause‐Specific Quality of Life (MENQOL),^14^ and the Korean version of the Patient‐Reported Outcomes Measurement Information System 29 Profile v2.1 (K‐PROMIS‐29 V2.1).^15^ Known‐group analysis was performed to demonstrate the questionnaire's ability to discriminate between two groups that differ on the variable of interest.^16^ The content of the improved text is as close as possible to the source text, and no new aspects have been added. A literature review was conducted to identify factors associated with the quality of life in endometrial cancer patients, including age, ECOG performance scale, comorbidities, and treatment status.^17^, ^18^, ^19^

### 2.4 Statistical analysis

For analysis of the individual EnCS items, interview field notes and transcripts were compiled, abstracted, and summarized item by item. The interview data were examined for semantic and conceptual equivalence to the original English items. The expert group reviewed the results of each round of interview data analysis. The proportion of respondents exhibiting any level of difficulty or hesitation for an item or response option was calculated. EnCS items that met the a priori threshold of ≥20% of respondents with comprehension difficulties in Round 1 were considered for rephrasing and retesting in Round 2. Items that at least 20% of the respondents found difficult to comprehend were classified under either linguistic or cultural difficulties. Item revision was considered on the basis of a detailed review of participants' responses and in the context of an effort to produce a final version that would be well comprehended by diverse respondents, including those who were older and had lower educational levels.

We performed exploratory factor analysis (EFA). To assess the internal consistency of the EnCS, we calculated the internal consistency of each domain using Cronbach's *α* and the item‐total correlation of each domain. An *α* value of 0.8 or higher indicates good reliability.^2^

We also performed confirmatory factor analysis (CFA) with the final items using the “maximum likelihood without missing values”.^20^ Comparative fit index (CFI) and standardized root mean squared residual (SRMR) were also calculated. A CFI > 0.9, SRMR < 0.08, and RMSEA < 0.06 indicate a good fit.^21^, ^22^

The test–retest reliability of the EnCS was measured using the intraclass correlation coefficient (ICC) under a two‐way mixed model. A questionnaire is considered reliable if the obtained ICC values are greater than 0.70.^23^ Using the repeated measure data set, the standard error of measurement (SEM) was calculated by first creating a variable for the difference between the score obtained during the first and second administrations.^24^ We then calculated the smallest detectable change (SDC), which was calculated by SEM × 1.96 × √2.^25^

To test the hypothesis for construct validity, we calculated convergent and discriminant validity using Pearson's correlations between the EnCS and the EORTC QLQ‐EN24, MENQOL, and K‐PROMIS‐29 V2.1. We expected EnCS had negative correlations with MENQOL (−0.70 ≤ *r* ≥ −0.30) and had positive correlation with EORTC QLQ‐EN24 and K‐PROMIS‐29 V2.1 as convergent validity (0.30 ≤ *r* ≥ 0.70).

*T*‐tests and ANOVA were used to determine if there are differences in the means of EnCS based on age, ECOG performance scale, comorbidities, and treatment status, in order to assess known‐group validity. If a statistically significant difference is found, it is confirmed to be valid.

All analyses were two‐sided, and *p*‐values <0.05 were considered statistically significant. Statistical analyses were performed using R software version 3.3.2 (Free Software Foundation, Inc., Boston, MA, USA).

## 3 RESULTS

### 3.1 Cognitive interview

A total of 11 women with endometrial cancer participated in the cognitive interview; 54.5% were over 60 years old and had lower literacy levels, with an education level of high school or lower. Although there were no linguistic or cultural difficulties, six out of eleven patients reported difficulty responding to the question “I have pain or discomfort with intercourse.” The reason for this was that the item was not relevant to them. However, this was because they were in the immediate postoperative period after hysterectomy, which prevented them from engaging in sexual intercourse due to potential dehiscence of the vaginal cuff. Therefore, we did not modify or change any item.

### 3.2 Study participants

In total, 240 patients with endometrial cancer participated in the survey. Their mean age was 54.3 (standard deviation 11.2) years. (Table 1) Although all patients completed all the questionnaire, 42 (17.5%) did not respond to the item “I have pain or discomfort with intercourse,” because they were not sexually active during the past 7 days as we found during the cognitive interview.

**TABLE 1:** Characteristics of study participants (*N* = 240).

Characteristics	*N* (%)
	*N* = 240
Age, mean (SD)	54.31 (11.24)
<40	26 (10.8)
40–49	34 (14.2)
50–59	106 (44.2)
60–70	59 (24.6)
≥70	15 (6.2)
Marital status	
Married	162 (67.5)
Single	38 (15.8)
Divorced/separated	20 (8.3)
Widowed	12 (5.0)
Living with partner	8 (3.3)
Education	
Less than high school	21 (8.7)
High school graduate	96 (40.0)
More than college/university	123 (51.2)
Employment	
Homemaker	95 (39.6)
Employee/self‐employed	92 (38.3)
Sick leave/retired	38 (15.8)
Unemployed	14 (5.8)
Other (e.g., student)	1 (0.4)
Household yearly income	
<$20,000	32 (13.3)
$20,000–$49,999	87 (36.2)
$50,000–$99,999	84 (35.0)
≥$100,000	37 (15.4)
ECOG performance scale	
0	153 (63.7)
1	36 (15.0)
2	23 (9.6)
3	28 (11.7)
Comorbidities, yes	151 (62.9)
Treatment status	
Pre‐treatment	7 (2.9)
On‐going treatment	132 (55.0)
Post‐treatment less than 1 year	28 (11.7)
Post‐treatment more than 1 year	73 (30.4)

Abbreviation: ECOG, European Cooperative Oncology Group.

### 3.3 Validity and reliability

All 16 items satisfied the Bartlett's test for sphericity (*p* < 0.01) and the Kaiser–Meyer–Olkin (KMO) test for sampling adequacy (*p* = 0.84). All the items had generally acceptable levels of item correlation (≥0.40) in EFA (Table 2). In four‐factor solution, the first factor included stomach‐related symptoms, including “swelling” “cramps” and “discomfort/pain” in the stomach area. The second factor included vagina‐related symptoms. The third factor included menopausal symptoms. The fourth factor included other general symptoms. While all the items had generally acceptable levels of item correlation (≥0.40), the item “In the past 7 days, I have pain or discomfort with intercourse” had a relatively low correlation (*r* = 0.36) with other items. We excluded the item “I have pain or discomfort with intercourse” in the confirmatory factor analysis (CFA).

**FIGURE 1:**
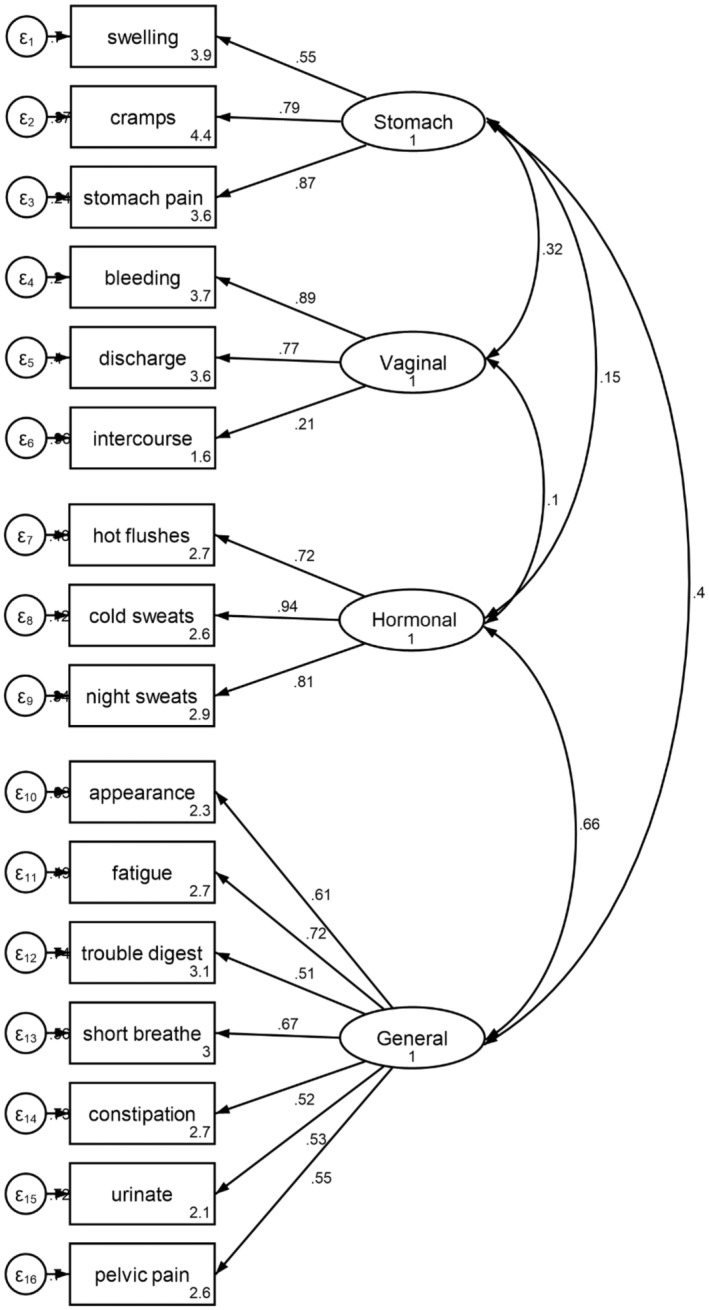
Confirmatory factor analysis of the endometrial cancer subscale (EnCS) in FACT‐EN.

The fit indices for the four‐factor solution model were good: CFI = 0.659; SRMR = 0.066, and RMSEA = 0.073 (Figure 1). In general, all the items had a small residual. However, “In the past 7 days, I have pain or discomfort with intercourse,” had a relatively large residual in both models.

**TABLE 2:** Factor loadings from the exploratory factor analysis of the endometrial cancer subscale (EnCS) in FACT‐EN (*N* = 240).

Items	1‐Factor solution	4‐Factor solution			
		Hormonal	Stomach related	General	Vaginal related
I have swelling in my stomach area	0.47		**0.55**		
I have cramps in my stomach area	0.48		**0.74**		
I have discomfort or pain in my stomach area	0.46		**0.87**		
I have vaginal bleeding or spotting	0.42				**0.81**
I have vaginal discharge	0.42				**0.77**
I am unhappy about a change in my appearance	0.64		0.36	**0.40**	
I have hot flashes/hot flushes	0.59	**0.66**			
I have cold sweats	0.68	**0.94**			
I have night sweats	0.67	**0.75**			
I feel fatigued	0.70	**0.49**		**0.47**	
I have pain or discomfort with intercourse	0.27			0.33	
I have trouble digesting food	0.45			**0.49**	
I have been short of breath	0.59	0.38		**0.50**	
I am bothered by constipation	0.45			**0.70**	
I urinate more frequently than usual	0.49			**0.50**	
I have discomfort or pain in my pelvic area	0.54		0.39	0.39	

*Note*: Bold values mean they are generally acceptable levels of item correlation (≥0.40).

The possible scores ranged from 0 to 172. The mean (SD) of total score of FACT‐EN was 122.84 (23.58). The floor and ceiling effects were 0.4% and 0.4%, respectively. Cronbach's *α* coefficients for the five scales of the EnCS ranged from 0.78 to 0.91 (Table 3). Cronbach's *α* coefficients of total score was 0.93 (Table 3).

**TABLE 3:** Description and statistics for FACT‐EN (*N* = 240).

Subscales	Range	Mean (SD)	% Floor	% Ceiling	Cronbach alpha
Physical well‐being	0–28	21.93 (5.93)	0.4	20.8	0.89
Social/family well‐being	0–28	18.01 (5.62)	1.7	3.8	0.81
Emotional well‐being	0–24	16.16 (4.67)	0.8	2.1	0.78
Functional well‐being	0–28	17.41 (6.32)	0.4	5.8	0.91
Total FACT‐G	0–108	73.51 (16.48)	0.4	0.8	0.91
Endometrial cancer subscale	0–64	49.32 (9.64)	0.4	3.8	0.85
Total FACT‐EN	0–172	122.84 (23.58)	0.4	0.4	0.93

Abbreviations: FACT‐EN, Functional Assessment of Cancer Therapy‐Endometrial Cancer; FACT‐G, Functional Assessment of Cancer Therapy‐General.

In the test–retest reliability (*N* = 90), the ICC of EnCS was 0.76 (95% CI: 0.62–0.84) which was a satisfactory consistency. The SEM of the EnCS score was 4.52, and SDC was 8.87.

The convergent and discriminant validity of EnCS had a moderate to high correlation with EORTC QLQ‐EN24 (range of *r*, −0.54 to −0.35) except sexual interest, sexual activity, and hair loss (*r* = 0.05, −0.01 and −0.23, respectively) and MENQOL (range of *r*, −0.71 to −0.30) (Table 4). In addition, EnCS had moderate to high correlation with K‐PROMIS‐29 V2.1 (range of *r*, −0.63 to 0.52, Table 4).

**TABLE 4:** Convergent and discriminant validity of FACT‐EN with legacy measures.

Legacy measures	EnCS	Total	PWB	SWB	EWB	FWB
EORTC QLQ‐EN24						
Lymphoedema	−0.37**	−0.42**	−0.30**	−0.23**	−0.14*	−0.20**
Urological	−0.53**	−0.53**	−0.34**	−0.20**	−0.28**	−0.23**
Gastrointestinal	−0.49**	−0.49**	−0.47**	−0.15*	−0.31**	−0.28**
Sexual interest	0.05	0.04	0.09	−0.05	0.11	0.1
Sexual activity	−0.01	−0.02	0.05	−0.05	0.07	0.08
Poor body image	−0.52**	−0.53**	−0.45**	−0.13*	−0.37**	−0.31**
Back/pelvic pain	−0.54**	−0.57**	−0.43**	−0.24**	−0.36**	−0.24**
Tingling/numbness	−0.35**	−0.42**	−0.37**	−0.15**	−0.22**	−0.27**
Muscular/joint pain	−0.38**	−0.44**	−0.37**	−0.19**	−0.27**	−0.23**
Hair loss	−0.23**	−0.25**	−0.27**	−0.08	−0.01	−0.13*
Taste changes	−0.39**	−0.47**	−0.52**	−0.07	−0.22**	−0.31**
MENQOL						
Vasomotor	−0.64**	−0.56**	−0.34**	−0.25**	−0.28**	−0.30**
Psychosocial	−0.70**	−0.79**	−0.58**	−0.35**	−0.54**	−0.57**
Physical	−0.71**	−0.73**	−0.57**	−0.32**	−0.41**	−0.43**
Sexual	−0.30**	−0.23**	−0.12	−0.07	−0.15	−0.10
Total	−0.76**	−0.76**	−0.56**	−0.38**	−0.44**	−0.48**
PROMIS‐29						
Physical function	0.41**	0.54**	0.53**	0.17**	0.30**	0.48**
Anxiety	−0.63**	−0.71**	−0.49**	−0.26**	−0.67**	−0.50**
Depression	−0.63**	−0.76**	−0.56**	−0.31**	−0.66**	−0.57**
Fatigue	−0.67**	−0.72**	−0.66**	−0.22**	−0.48**	−0.50**
Sleep disturbance	−0.37**	−0.45**	−0.27**	−0.21**	−0.29**	−0.48**
Ability to social role	0.52**	0.65**	0.61**	0.23**	0.36**	0.58**
Pain interference	−0.54**	−0.68**	−0.67**	−0.20**	−0.47**	−0.57**

Abbreviations: EnCS, endometrial cancer subscales; EWB, emotional well‐being; FWB, functional well‐being; PWB, physical well‐being; SWB, social well‐being.

**p < 0.01; * *p* < 0.05.

In the group analysis, older age and lower ECOG performance scores were associated with higher EnCS scores (Table 5). When comparing the subdomain scores, it was observed that the stomach and vaginal domains in EnCS were higher in patients who had completed treatment for more than 1 year compared to those who were still undergoing treatment.

**TABLE 5:** Endometrial cancer subscale (EnCS) by characteristics of study participants.

Characteristics	Overall EnCS adjusted^a^ difference (95% CI)	Hormonal domain in EnCS adjusted^a^ difference (95% CI)	Stomach domain in EnCS adjusted^a^ difference (95% CI)	General symptom domain in EnCS adjusted^a^ difference (95% CI)	Vaginal domain in EnCS adjusted^a^ difference (95% CI)
Age group					
<40	Reference	Reference	Reference	Reference	Reference
40–49	1.43 (−3.41, 6.27)	0.48 (−1.04, 1.99)	0.58 (−0.46, 1.62)	−0.22 (−2.86, 2.42)	0.59 (−0.65, 1.82)
50–59	2.33 (−1.41, 6.48)	0.38 (−0.92, 1.68)	**0.98 (0.09, 1.87)**	0.31 (−1.96, 2.57)	0.84 (−0.21, 1.89)
60–70	**6.47 (1.99, 11.00)**	1.31 (−0.10, 2.71)	**1.40 (0.44, 2.37)**	**2.58 (0.13, 5.03)**	**1.32 (0.17, 2.48)**
≥70	2.60 (−3.51, 8.70)	1.36 (−0.56, 3.27)	1.10 (−0.21, 2.41)	−0.47 (−3.80, 2.86)	**1.85 (0.01, 3.68)**
ECOG performance scale					
0	**5.21 (0.94, 9.48)**	**1.34 (0.00, 2.68)**	**1.91 (0.99, 2.83)**	1.92 (−0.41, 4.25)	0.12 (−1.04, 1.28)
1	4.40 (−0.22, 9.02)	0.33 (−1.12, 1.78)	**2.16 (1.17, 3.16)**	2.23 (−0.29, 4.25)	0.06 (−1.15, 1.27)
2	3.04 (−2.09, 8.17)	1.26 (−0.35, 2.87)	1.10 (−0.00, 2.20)	1.02 (−1.78, 3.82)	0.31 (−1.08, 1.70)
3	Reference	Reference	Reference	Reference	Reference
Comorbidities					
No	2.29 (−0.26, 8.17)	0.76 (−2.03, 2.61)	0.04 (−0.51, 0.59)	**1.69 (0.30, 3.08)**	2.32 (−0.24, 4.88)
Yes	Reference	Reference	Reference	Reference	Reference
Treatment status					
Pre‐treatment	5.05 (−2.35, 12.50)	0.29 (−2.03, 2.61)	0.77 (−0.82, 2.36)	**4.06 (0.02, 8.10)**	0.98 (−1.02, 2.98)
On‐going treatment	Reference	Reference	Reference	Reference	Reference
Post‐treatment less than 1 year	1.69 (−2.49, 5.87)	−0.18 (−1.49, 1.13)	0.56 (−0.34, 1.46)	0.59 (−1.69, 2.87)	0.91 (−0.35, 2.18)
Post‐treatment more than 1 year	2.38 (−0.88, 5.64)	−0.60 (−1.62, 0.43)	**0.99 (0.29, 1.69)**	1.11 (−0.67, 2.88)	**0.88 (0.01, 1.74)**

*Note*: Bold values mean they are statistically significant values.

Abbreviations: ECOG, European Cooperative Oncology Group; EnCS, Endometrial Cancer Subscale.

^a^ Adjusted for all covariates in Table 5.

## 4 DISCUSSION

The present study demonstrated that the FACT‐EN is a valid and appropriate tool for assessing quality of life (QoL), symptoms, and function of patients with endometrial cancer. The EFA revealed better performance with the inclusion of four factors although the EnCS is originally a single domain with total score. The CFA using a four‐factor solution yielded good results. Regarding convergent and discriminant validity, the EnCS had a strong or moderate correlation with the MENQOL and EORTC QLQ‐EN24. The EnCS showed satisfactory consistency.

According to a recent systematic review, the EORTC QLQ‐C30 questionnaire was found to be the most commonly used instrument in EC studies.^6^ This questionnaire includes a global health status/quality of life indicator and various subscales, such as functional scales, and cancer‐related symptoms.^26^ However, despite their clinical importance for patients, lymphedema, gastrointestinal and genitourinary symptoms, and sexuality have been inadequately evaluated in EC survivors due to lack of appropriate instruments.^3^ On the other hand, the EnCS assesses the functions and symptoms commonly experienced by endometrial cancer patients. We identified the EnCS consisted of four factors: stomach‐related, menopausal‐related, vaginal‐related, and general symptoms. Similar result previously published which have a five‐factor solution for symptoms in EFA of FACT‐EN, including gastrointestinal, hormonal, psychological, respiratory, and weight change.^9^ Each issue addressed unique features, and the problem areas varied among patients. It is necessary to recognize the presence of items, considering that different problems may require distinct solutions or supportive care.^10^

In fact, different patients had different problem in the known‐group analysis. According to a previous cohort study, the QoL of women with advanced gynecological cancer appears to improve over time with regard to physical functioning, physical role function, bodily pain, and vitality.^27^ However, we found that the hormone‐related domains did not show recovery. Menopause can also be induced by endometrial cancer treatments. Surgical removal of the ovaries in premenopausal women can lead to premature or early menopause. Pelvic radiotherapy and certain chemotherapy treatments can also cause ovarian failure. Anti‐endocrine therapy, administered for hormone‐sensitive malignancies, can induce vasomotor symptoms.^28^ Iatrogenic menopause may be more severe and long lasting than physiological menopause.^29^ Following a cancer diagnosis, women may be at an increased risk of affective disorders such as depression and anxiety, and menopausal symptoms such as sleep disturbance may exacerbate this risk.^28^ Therefore, it is necessary to consider the subdomain scores instead of relying solely on the total score. Furthermore, it is important to include endometrial‐specific symptoms during assessments.

Furthermore, we confirmed that each factor in the EnCS can be effectively measured using valid measurement tools. In terms of the convergent and discriminant validity of the EnCS, the EORTC QLQ‐EN24 demonstrated a strong correlation with the EnCS. However, EORTC QLQ EN24 did not include menopausal symptoms, which is a frequent problem in patients with endometrial cancer. In this study, we confirmed that the EnCS was strongly correlated with the MENQOL, which specifically covers menopause‐related symptoms. Thus, the FACT‐EN is beneficial in evaluating comprehensive QoL in patients with endometrial cancer. The QoL measuring FACT‐EN could be important disease‐specific outcomes to this patient population.

### 4.1 Study limitations

A limitation of this study is that we recruited individuals visiting an outpatient clinic at only one institution in South Korea. These findings may not be generalizable to patients in other settings. However, this study included patients with low income and various survival times. In addition, we included patients with very low educational attainment, suggesting that it has acceptable measurement properties for use among diverse patients with cancer. Another limitation is that the item, “pain or discomfort with intercourse,” had a relatively large residual. In a recent study, 55.9% of endometrial cancer survivors never engaged in sexual intercourse with their partners after surgery.^30^ Although clinicians recommend temporal restriction of sexual activity after treatment, patients often held a misconception about permanently abstaining from sexual intercourse after undergoing cancer treatment.^31^ Thus, it is necessary to inform patients that the question aims to assess whether they abstain from sexual activity.

### 4.2 Clinical implications

In EC, numerous unanswered questions regarding patient care remain. It is crucial to identify important QoL and patient‐reported outcome questions early on, clearly state the hypotheses, and select the appropriate instruments. In this study, the FACT‐EN has demonstrated its validity as an assessment tool with significant implications for capturing various symptoms in patients with endometrial cancer. In this regard, the FACT‐EN holds great value as a potentially valuable option among available instruments.

## AUTHOR CONTRIBUTIONS

**Sooyeon Kim:** Conceptualization (equal); data curation (lead); formal analysis (lead); methodology (equal); writing – original draft (lead). **Joseph J. Noh:** Conceptualization (equal); methodology (equal); resources (lead); writing – original draft (equal). **Youngha Kim:** Conceptualization (equal); methodology (equal). **Juhee Cho:** Conceptualization (equal); methodology (equal). **Danbee Kang:** Conceptualization (equal); formal analysis (supporting); methodology (equal); writing – review and editing (lead). **Yoo‐Young Lee:** Conceptualization (equal); methodology (equal); resources (lead); writing – review and editing (equal).

## CONFLICT OF INTEREST STATEMENT

The authors have none to declare.

## Supporting information


Tables S1–S2

## Data Availability

The data underlying this article are available in the article.
