# Supplementary material for: Psychometric validation of the Functional Assessment of Cancer Therapy‐Endometrial among endometrial cancer patients
Source: Cancer Med. 2024 Mar 11;13(5):e7096. doi: 10.1002/cam4.7096 (PMC10926879; doi:10.1002/cam4.7096)
Supplement: Supplementary file 1 — Tables S1–S2 [file CAM4-13-e7096-s001.docx]

**Supplementary Table 1.** Characteristics of study participants (N = 240)

| **Characteristics** | | **N (%)** |
| --- | --- | --- |
|  |  | **N = 240** |
| **Age, *mean (SD)*** | | 54.31 (11.24) |
|  | < 40 | 26 (10.8) |
|  | 40-49 | 34 (14.2) |
|  | 50-59 | 106 (44.2) |
|  | 60-70 | 59 (24.6) |
|  | ≥ 70 | 15 (6.2) |
| **Marital status** | |  |
|  | Married | 162 (67.5) |
|  | Single | 38 (15.8) |
|  | Divorced/separated | 20 (8.3) |
|  | Widowed | 12 (5.0) |
|  | Living with partner | 8 (3.3) |
| **Education** | |  |
|  | Less than high school | 21 (8.7) |
|  | High school graduate | 96 (40.0) |
|  | More than college/university | 123 (51.2) |
| **Employment** | |  |
|  | Homemaker | 95 (39.6) |
|  | Employee/self-employed | 92 (38.3) |
|  | Sick leave/retired | 38 (15.8) |
|  | Unemployed | 14 (5.8) |
|  | Other (e.g., student) | 1 (0.4) |
| **Household yearly income** | |  |
|  | < $20,000 | 32 (13.3) |
|  | $20,000-$49,999 | 87 (36.2) |
|  | $50,000-$99,999 | 84 (35.0) |
|  | ≥ $100,000 | 37 (15.4) |
| **ECOG performance scale** | |  |
|  | 0 | 153 (63.7) |
|  | 1 | 36 (15.0) |
|  | 2 | 23 (9.6) |
|  | 3 | 28 (11.7) |
| **Comorbidities, yes** | | 151 (62.9) |
| **Treatment status** | |  |
|  | Pre-treatment | 7 (2.9) |
|  | On-going treatment | 132 (55.0) |
|  | Post-treatment less than 1 year | 28 (11.7) |
|  | Post-treatment more than 1 year | 73 (30.4) |

ECOG, European Cooperative Oncology Group

**Supplementary Table 2.** Description and statistics for FACT-EN (N = 240)

| **Subscales** | **Range** | **Mean (SD)** | **% Floor** | **% Ceiling** | **Cronbach alpha** |
| --- | --- | --- | --- | --- | --- |
| Physical well-being | 0-28 | 21.93 (5.93) | 0.4 | 20.8 | 0.89 |
| Social/family well-being | 0-28 | 18.01 (5.62) | 1.7 | 3.8 | 0.81 |
| Emotional well-being | 0-24 | 16.16 (4.67) | 0.8 | 2.1 | 0.78 |
| Functional well-being | 0-28 | 17.41 (6.32) | 0.4 | 5.8 | 0.91 |
| **Total FACT-G** | 0-108 | 73.51 (16.48) | 0.4 | 0.8 | 0.91 |
| Endometrial cancer scale | 0-64 | 49.32 (9.64) | 0.4 | 3.8 | 0.85 |
| **Total FACT-EN** | 0-172 | 122.84 (23.58) | 0.4 | 0.4 | 0.93 |

FACT-G: Functional Assessment Cancer Therapy-General; FACT-EN: Functional Assessment Cancer Therapy-Endometrial Cancer
